# Treatise on the Excision of Diseased Joints

**Published:** 1831

**Authors:** 


**TREATISE ON THE EXCISION OF DISEASED JOINTS. By *James Syme*, Esq. F.R.S.E., Surgeon of the Edinburgh Surgical Hospital, Lecturer on Surgery, &c. &c. 8vo. pp. 163. Edinburgh, Adam Black, 1831.**

THOSE who have paid attention to the progress of modern surgery, will acknowledge that its advance has been by two different paths: in the performance of operations formerly unheard of, or considered impracticable; and, in the non-performance of operations formerly of very frequent occurrence. Limbs were amputated for diseases some twenty or thirty years ago, which are commonly curable without amputation now, and we think that this constitutes the truest, though the least obtrusive, glory of our science. The proper object of medicine and surgery is the alleviation of human woe, and this we conceive is better attained by dispensing, if possible, with the knife altogether, than by using it with the most consummate dexterity and success. Amputations have always been regarded with some feelings of disgust; it is a humiliating as well as a painful spectacle to see a limb lopped off for disease; it is, as has been justly remarked, a confession of our inability to cure, of the incapacity of our science. Any proposal, then, intended to mitigate the severity of this mutilation, should be candidly received and carefully examined. Undoubtedly its merits should not be blindly and hastily admitted, but neither should they be dogmatically nor obstinately denied. It is a pleasant and besetting sin in human nature, to refer the *cause* of all innovations to the arbitrary decisions of prejudice and habit.

For many years past a few surgeons have been successively struggling to drag into notice excision of some joints as a substitute on some occasions for amputation. As yet they have scarcely succeeded, but latterly the publication of cases in the medical journals has drawn a considerable degree of attention to the subject. Our readers need scarcely be told that Mr. Syme has rather distinguished himself in this department; that, in fact, he has become the Coryphæus of the excising party in this country. He has previously given to the world detached papers and cases in reference to the question, and now he steps forward in the dignified garb of an octavo. As we believe that the surgical part of the profession are beginning to direct their serious attention to the advantages or otherwise of excision, we cannot do better than lay the substance of the work before them. As usual we shall analyze.

The first chapter which treats of the diseases of joints is not worth notice, but the second, in which are discussed the relative merits of excision and amputation, must give us pause. The following are our author's remarks on excision.

"The great recommendation of excision is, that it saves the patient's limb; and the benefits accruing to him from this are so important and conspicuous, that, unless the objections which can be urged against it should appear after mature consideration to be very serious indeed, we ought not to hesitate in giving it the preference. These objections, so far as I have been able to ascertain, are the following :—*First,* The difficulty of the operation. *Second,* Its danger. *Third,* The useless condition of the limb in which it has been performed.

In taking into consideration the difficulty of the operation, it must be ascertained, in the first place, what is requisite to constitute its effectual performance : in other words, how far it is necessary to take away the diseased integuments, synovial membrane, articulating cartilage, and extremities of the bones. In cases of old standing, where the sinuses are numerous and the suppuration has been profuse, the integuments surrounding the joint often retain hardly any trace of their original appearance or structure. They lose their laxity and mobility, from effusion of lymph into the subjacent cellular substance ; become smooth and shining on the surface, which is often of a dark-red or purple colour ; and are so soft, that, if stitches are introduced to approximate the edges of an incision made in them, the threads instantly cut their way out. It might, therefore, be supposed that no healthy union or permanent cure could be obtained if parts in such a morbid state were allowed to remain, and that, consequently, the operation could very seldom be practised with propriety. Experience, however, has shown that this is not the case ; and that in a very few days after the operation, when the swelling and inflammation immediately consequent upon it begin to subside, the diseased integuments regain their natural characters, and ultimately become perfectly sound.

As to the synovial membrane, Mr. Brodie has stated his opinion, that, when once its structure has been completely altered, it cannot be restored.* Independently of his high authority, it might be readily believed, that, if any of the thick gelatinous substance into which this membrane is transformed were permitted to remain, a cure could hardly be accomplished ; and that, as this portion of the articular apparatus is not only very extensive, but likewise most intimately connected with the surrounding tissues, it must consequently be next to impossible to perform the operation of excision effectually. Experience here also, however, has decided the matter to be otherwise ; and it is proved beyond dispute, by the facts hereafter to be mentioned, that the synovial membrane, though thickened and gelatinized to the utmost, affords very little obstacle to recovery, since it speedily disappears, partly by sloughing, but chiefly through the absorbent action of its own vessels, during the copious suppuration which ensues.

With regard to the cartilage, it might be expected that no harm could result from leaving any part of it that remained sound ; but here, too, the judgment of theory is reversed by experience, since it has been found, that, when any portion of the articulating surface was left, the disease required a subsequent operation. The cause of this is probably to be referred, not so much to any morbid process in the cartilage itself, as in the synovial membrane lining it, and in the spongy bone immediately subjacent, which has its tendency to morbid action excited by the injury sustained in its neighbourhood. The operation, therefore, essentially requires the removal of the whole cartilaginous surface.

Lastly, as to the bone, one not acquainted with the pathology of the osseous tissue, who examined the bones of carious joints after maceration, might be apt to suppose that the diseased part could not be removed without sacrificing so large a portion of the whole, as to render it useless and unworthy of preservation. Plate I. Fig. 1, will illustrate this. The bones represented here are those of an elbow-joint, which I amputated before adopting the plan of treatment now under consideration. It will be observed that they are much increased in thickness to a considerable distance from the articulation, and that their surface in the whole of this extent is covered with irregular warty excrescences, which give it a rough tubercular appearance. When these tubercles are examined more particularly, they are found to consist of a compact osseous sub-

* Vid. Op. et loc. cit.

stance, which is smooth on the surface, and perforated with numerous apertures for the transmission of blood-vessels. This is new bone, and perfectly healthy in its actions; it resembles in all respects the callus, or new osseous substance, which effects the reparation of fractures, and is thrown out in consequence of the irritation of the disease. The truly morbid or carious portion of the bone is seen between the lines AA and BB, occupying merely the articulating surfaces. The external shell of the spongy bone is here removed by the disease, and the cancelli are exposed to view, presenting a rough surface composed of rigid plates and spiculæ, which are white and more brittle than usual, so as to seem as if they had been subjected to the action of fire. The depth to which the bone is thus affected varies considerably, according to the origin of the disease. When the morbid action commences in the synovial membrane or cartilage, it is generally superficial; but when the inflammation is primarily seated in the substance of the spongy bone, as in the third kind of white-swelling which has been mentioned, then, as has been already stated, the substance of the bone is more deeply affected, being often excavated into a hollow, which is carious over the whole of its surface. The extent of this cavity seldom, or rather never, exceeds the bounds of the epiphyses, except sometimes in young subjects, where the bone has been widely altered by scrofulous action, previous to suffering the inflammation which more immediately occasions the caries. From not distinguishing between the truly diseased bone and that effused in consequence of its irritation, it appears that a much larger portion has been taken away in some of the cases of excision hitherto published than there was any occasion for. Less than a half of the portions of the humerus and femur which were removed by Moreau and Crampton, I should certainly think, so far as can be judged from the evidence of their drawings, would have been sufficient for the purpose, in which case it is plain the limbs would have been much less shortened and weakened, and the magnitude and consequent severity of the operation diminished. As already stated, the caries seldom goes beyond the epiphyses, which are all the part of the bone that the surgeon requires to remove, except in the rare cases where the bone is found to be more extensively affected; and in these it will probably be most prudent to perform amputation." 22.

From this analysis Mr. Syme comes to the conclusion, that all essentially required in the operation is the removal of the articulating epiphyses. With regard to the means of performing it, Mr. S. gives the preference to Mr. Liston's cutting plyers, whenever the ordinary saw is not applicable. The difficulties of the operation are certainly sometimes considerable; but they are such as can be surmounted by a moderate share of dexterity and coolness. The danger of excising a diseased joint is, for obvious reasons, not to be compared with that of opening into a sound one. Patients have been observed to sleep better the night after the operation than for a long time previously. From a consideration of all the circumstances Mr. S. thinks it not unreasonable to conclude that the danger from amputation is greater than from excision, and this conclusion appears to be borne out by facts. Mr. S. has cut out fourteen elbow-joints, and the operation has been performed in Edinburgh three times by other practitioners. Of these seventeen cases two only have terminated fatally, and in one Mr. Syme believes that the patient would have died from any operation, while in the other the disease was found so extensive as to render the excision almost impracticable. He believes that the result of seventeen amputations in similarly unfavourable constitutions would not be so satisfactory. Here we cannot refrain from remarking that the operation of excision appears to be too frequently performed in Edinburgh. At a large hospital in London we have not witnessed seventeen amputations for diseased elbow in the course of many years, and yet we need hardly say that great numbers of such cases have been treated at the institution. The majority of patients thus affected have done well without any operation. They have had more or less anchylosis it is true, but still we presume that the warmest advocates for excision will scarcely prefer its risks and consequences to anchylosis without an operation at all. We cannot but suspect that joints are excised by Mr. Syme and others, which might be saved by time and perseverance.

The last point to be considered is the subsequent state of the limb. Experience has proved that true anchylosis or osseous union does not even frequently occur, indeed not without very great attention on the part of both the surgeon and patient, and particularly not without absolute rest. When no such precautions are employed, the union is by means of a tough, flexible, ligamentous-like substance, that permits the bones to be used with more or less freedom, according to the exercise which they are made to undergo during the process of healing. The voluntary motion, at first impaired or altogether lost, gradually returns, and ultimately becomes as strong as ever. Mr. Syme winds up by asserting, that the cases to be related will prove that it is possible to save limbs by excision of diseased joints nearly, if not altogether, as useful as before they suffered from disease.

### Method of performing Excision of the Joints.

The precepts given by Mr. Syme are these:—Let the patient be placed in the position which most readily exposes the articulation, and is preserved with least difficulty. A tourniquet is seldom necessary. The most convenient scalpel is a long, narrow one, straight in the back, very slightly convex in the edge, stoutly made, having a small part of the back ground off obliquely at the point, so as to render it less apt to be broken, and to bring it to correspond with the axis of the handle. The preliminary incisions should be free; in making them, the knife should be thrust at once into the joint, and afterwards carried close down to the bones, which shortens the operation, lessens pain, and renders the line of incision more determined in its direction. The muscular and tendinous parts should be as little injured as possible, and the most effectual method of saving them is to cut them close away from their attachment. The saw may be either a simple blade, or the one in common use for amputation, which Mr. S. believes to be the most convenient. He finds the hand the most effectual guard for the defence of the soft parts; and, instead of cutting the bone completely through with the saw, it is often better to divide it only partially with that instrument, and then resort to the cutting pliers, which readily detach the fragment as soon as there is a groove formed for the reception of their blades. Unless the bone is very large and hard, the pliers are of themselves sufficient for the purpose. The flat sides of the blades should be turned towards that surface of the bone which is to remain, as it will thus be less apt to be splintered or irregular.

"When all the diseased bone has been got away, which will be learned by a careful examination of the separated fragments and the remaining surface, if there are any large masses of gelatinous substance which can be easily detached, it is as well to remove them, since, though they would not afford any great obstacle to recovery, they might have some effect in retarding it, and also preventing the edges of the wound from coming readily together. Though the hemorrhage is generally pretty free in the first instance, it seldom persists so as to require the application of ligatures. The general oozing from the surface is usually soon checked by exposure to the air, or washing with cold water; but if, after the operation is ended, one or more arteries should continue to throw out a jet, they ought to be secured, as it is next to impossible to exert pressure with any effect, and considerable inconvenience is apt to result from the cavity becoming distended with blood. The vessels that prove obstinate are generally situated in the indurated subcutaneous cellular tissue, and require considerable care both for their discovery and ligature.

The next part of the process is to place the edges of the wound in contact, and retain them together, which is best effected by the interrupted suture, unless the integuments should be so very soft as to give way under the pressure of the threads, in which case compresses of lint must be used in their stead. It is always of most consequence to unite the edges of the transverse incision, if there is one, since, if they do not heal by the first intention, they are afterwards brought together with very great difficulty, and the broad cicatrix which results from their separation is very adverse to the mobility of the joint. Some compresses of lint ought to be applied over the flaps, and then the limb being placed in a proper position, that, namely, in which it will most frequently be required after the cure is completed, it ought to be enveloped with a long roller, which affords the requisite support much better than splints or rigid cases of tin or pasteboard.

The constitutional disturbance, for the reasons already stated, is usually very slight, and requires nothing more than some gentle purgative or slight antimonial, with spare diet and rest. The pain is usually severe for the first five or six hours, but then subsides, and seldom proves troublesome afterwards. The dressings ought to be changed ten or twelve hours after the operation, by which time the oozing of blood and serum will be at an end; and then also any inequality or gaping of the edges may be rectified by slips of sticking-plaster. Union by the first intention sometimes takes place through nearly the whole line of incision, except where old sinuses exist in its course; more frequently the adhesion is only partial, and the wound opens out more or less widely, according to the degree of local inflammation, and the distention caused by blood contained within its cavity. In the course of a few days, the discharge, which was at first copious and offensive, begins to diminish; all the clots of blood issue from the wound; the swelling subsides; and the favourable change is altogether so sudden and satisfactory, as to surprise those who are not accustomed to witness the operation.

During the cure every means is to be employed either to keep the limb perfectly quiet, to favour anchylosis, or to exercise it in the degree and extent of mobility which will be required of it. The wound is generally very nearly healed in the course of a few weeks, but one or more sinuses continue to discharge for months, or even a year or two. Small portions of bone also occasionally come away; but if the surgeon has done his duty in the first instance, he need not be under any apprehension on these accounts; and the patient will be too well pleased with being freed from the pain of his disease, and having regained the use of his limb, to feel annoyed by the trifling inconvenience which he thus experiences." 37.

### Excision of the Shoulder-joint.

Having noticed the preceding part of the volume, which treats of excision of joints generally, we shall endeavour to be brief with the succeeding portion, which is devoted to the consideration of the particular articulations. We are induced to do this because much has already been published on the subject, and those who wish for very particular information may readily refer to Mr. Syme's book, his former papers, or to the article on Excision of Joints in the Surgical Dictionary.

The humerus is not always affected to the same extent, but all that part above the attachments of the pectoralis major and latissimus dorsi should be taken away, and that in the first instance, to afford room for getting access to the scapular part of the disease. The acromion sometimes participates in the disease, and then requires removal. By opening the articulation at its external or lateral part, and then cutting close to the bones the axillary artery is perfectly safe. The only vessel requiring a ligature is the posterior circumflex artery.

The best way of bringing the bones completely within reach, with least injury to the soft parts, is to make a perpendicular incision from the acromion through the middle of the deltoid, nearly to its attachment, and then another shorter one upwards and backwards, from the lower extremity of the former, so as to divide the external part of the muscle. The flap being dissected off, the joint will be brought into view, and the capsular ligament, if still remaining, having been divided, the finger may be passed round the head of the bone, so as to feel the attachments of the spinati and subscapular muscles, which can then be readily divided by introducing the scalpel, first on one side and then upon the other. The elbow then being pulled across the fore-part of the chest, the head of the humerus will be protruded, and can be easily sawed off whilst grasped in the operator's left hand. The subsequent part of the operation is to be conducted on the principles already laid down. As the preservation of as much mobility as possible is desirable, motion should be restrained no further than is necessary to prevent irritation and displacement. The tendency of the pectoralis major and latissimus dorsi to draw the extremity of the bone inwards may be easily prevented by placing a cushion in the axilla. Two cases are detailed; we shall shortly notice the first.

*Case.* Christian Laing, æt. 38, applied to Mr. Syme in June, 1825. She complained much of pain in the left shoulder, shooting down the arm to the fingers—the articulation was nearly immoveable, and the limb entirely useless, being constantly kept in a sling. There was a small opening directly under the acromion, and another about the middle of the clavicular part of the pectoralis major, both of which allowed the probe to pass deeply in the direction of the joint. The discharge was thin and copious, the integuments natural, and there was little swelling. She was a healthy-looking woman, apparently somewhat exhausted by anxiety and suffering. The disease had commenced six years previously after a fall upon the shoulder. For five years after the injury she suffered only pain and stiffness, but then, after some rough usage, an abscess formed on the fore-part of the joint, and, still more recently another. Mr. S. merely dilated the sinuses and sent the patient into the country. In the following March she returned, much thinner and weaker than before, with another sinus just above the posterior margin of the axilla, an almost incessant gnawing pain, little appetite, and an exhausting diarrhœa. Nothing but an operation promising relief, Mr.

Syme proposed to cut down upon the joint, and excise the bone or amputate the limb according to circumstances. His mode of operation having been already described, we need not go into the details in this place. The head of the humerus being hollowed into a cavity was removed; the glenoid cavity seemed sound, though divested of its cartilage; but the extremity of the acromion was bare and rough, and was cut away with the cutting-pliers. The operation occupied ten minutes. An attack of erysipelas followed the operation, but in a few weeks the wound was healed, except at those parts which corresponded with the openings of the previous sinuses. The use of the limb returned gradually, and by degrees the sinuses dried up, though one of them continued to afford a few drops of serous exudation for two years. She is now, four years and a half after the operation, in the following state. She is stout, active, and young-looking; manages her domestic concerns as a tradesman's wife; sews, knits and washes; and can carry a full pitcher of water with the left arm. There is no discharge or uneasiness of any sort. The left arm is about an inch shorter than the right. The shoulder, when examined bare, exhibits a deep, irregular, unseemly cicatrix. The joint allows the limb to be moved in all directions to nearly the natural extent, but the voluntary command over it is much more limited. She can move it across the chest, both backwards and forwards, with considerable force and freedom, but she has very little power of abduction.

In the other case of excision of the head of the humerus, there were symptoms of affection of the lungs before the performance of the operation, and the man died of phthisis six months after it.

### Excision of the Elbow-joint.

This is much more difficult, from the anatomical characters of the parts, than excision of the head of the humerus. The operation may be required either for caries, or for the effects of external injury both primary and secondary. The part that usually suffers most from caries is the olecranon, which is not unfrequently hollowed into a cavity, and diseased throughout; the radius and humerus are in general affected but superficially, and the disease very seldom extends beyond the head of the former and tuberosities of the latter. It is always right to take away the whole of the sigmoid cavity of the ulna, comprehending the olecranon and coronoid processes, together with the head of the radius and extremity of the humerus, as high as its tuberosities. The removal of more would be unnecessary, of less useless.

"The easiest way of accomplishing this is to remove the olecranon in the first place; then to cut the lateral ligaments of the joint, so as to free the extremity of the humerus, and saw it off; lastly, to detach, by means of cutting-pliers, the head of the radius, and the remaining part of the sigmoid cavity. The reason for not separating at once the whole of the ulna that requires to be removed is, that, in case it is divided below the insertion of the *brachiæus internus*, its removal becomes extremely difficult. Having experienced this inconvenience in one of my first cases, I have since always proceeded as has just been described, and never found any difficulty in detaching the coronoid process after gaining the free space that was afforded by removing the olecranon.

A simple longitudinal incision will not give sufficient access to the joint to allow of its excision, even in a sound state of the parts, much less when they are thickened and preternaturally adherent, as in cases of caries. An additional transverse cut was therefore proposed by Mr. Park, intersecting the other at right angles; but this plan labours under the double objection of splitting the triceps, and not permitting free exposure of the humerus. A method still more objectionable, on the ground of unnecessarily injuring the muscles, is to make a longitudinal incision, and two transverse ones at its extremities, so as to form two lateral flaps. By far the best plan that has yet been contrived is that of Moreau; and though it may appear at first sight complicated and destructive to the soft parts, it is really the easiest and least injurious that can be imagined. The figure H gives a perfect idea of his incisions; and it is only necessary to state further in explanation of them, that the transverse one should be close above the olecranon. In making this cut the ulnar nerve is apt to be wounded or divided; and though the facts mentioned below make this injury appear of very little consequence, as there can be no advantage in inflicting it, the surgeon ought to use the precaution of ascertaining the situation of the nerve before introducing his knife. The thickening of the limb is sometimes not so great as to prevent the nerve from being felt, but more frequently its situation can be discovered only by recollecting its position relatively to the bones; it lies close to the inner edge of the olecranon, and will certainly be cut if the transverse incision is prolonged farther than this towards the internal tuberosity of the humerus. The surgeon, therefore, ought to feel for the olecranon, and introduce his knife close to its upper surface, with the back turned towards its inner margin, but somewhat nearer its radial side. Having thrust the knife down into the joint, he ought to cut transversely, with a sawing motion, so as to insure the division of the tough tendinous parts, until he arrives at the radial tuberosity of the humerus. He may then make the longitudinal incisions, which should extend about an inch and a half upwards and downwards, without any danger whatever, as the oblique course of the nerve recedes from the line of division. Both flaps should be dissected previously to commencing the excision of the bones, as it is thus rendered much easier than when the exposure is confined to the part that is to be first removed. The hemorrhage is generally profuse immediately on the incisions being made, but soon diminishes, and seldom persists to such extent as to require the application of a ligature; on the principle already stated, however, it is right to secure any vessel, however small, that threatens to continue to bleed. In those rare and perplexing cases, where the ulna is diseased below the coronoid process, and requires to be divided through its shaft, the interosseous artery is very apt to be divided, and must, of course, be tied. As to the humeral artery, it is always perfectly safe, being protected from injury by the whole thickness of the *brachialis internus*." 70.

There is great variety in the difficulty experienced in different cases, owing to the nature of the adhesions, &c. After the operation the edges of the wound should be stitched together, the limb should be half bent, and a long roller applied in the figure of eight to give it proper support. The best position for the patient in cutting out the elbow, is lying with his face downwards on a sofa or table covered with a mattress.

Thirteen cases of excision of the elbow-joint are detailed. As many, if not most of them, have been formerly published in other journals, and some have been noticed on different occasions in this, we shall waive their consideration in the present place. We pass to—

### Excision of the Wrist-joint.

The construction of this joint, with its many bones and the numerous vessels and tendons in its vicinity, would seem to forbid, or, at all events, furnish strong arguments against excision. Mr. Syme has never performed the operation, but, if circumstances should render it advisable he would proceed in the following manner. Two longitudinal incisions, about an inch and a half in length, should be made from the extremities of the radius and ulna upwards along the lateral aspects of these bones. Then two shorter cuts are to be carried inwards on the posterior surface of the wrist, from the lower ends of the former ones. The extensors of the thumb will be divided, and the radial artery must be carefully avoided. The bones, being exposed as well as possible, should be divided with the pliers as high as necessary, when their removal will be easily accomplished, after which the carpal part of the articulation may be readily cut away with the pliers and gouge. It is said that Moreau, jun. and Roux have performed this operation with success.

In caries of the carpal bones, it is hardly prudent to attempt excision unless the disease is stationary, of limited extent, and without thickening in the neighbourhood. In operating, the surgeon should give himself plenty of room by a free crucial incision, and having exposed the bone by raising the flaps he may remove, with the gouge and pliers, the carious portion, which he recognizes by its softness. When the first joint of the fingers or thumb is affected, amputation is preferable to excision, as the shrunk and powerless digit is only an annoyance to the patient.

### Excision of the Hip-joint.

Reasoning would lead to the supposition that excision is not so applicable to the articulations of the lower extremities as to those of the upper. But we yet want sufficient facts to set the question entirely at rest. Excision of the hip-joint, for disease, has been proposed. Mr. Syme thinks that the almost invariable implication of the acetabulum in the ulcerative process is a sufficient bar to such an operation. Sometimes obstinate sinuses about the hip are owing to exfoliation of the bones of the pelvis, and the removal of the diseased portion is followed by a cure. Where the head of the thigh-bone has been shattered by a musket-bullet, without extensive injury of the soft parts, extraction of the fragments would be preferable to amputation. In trials on the dead subject, a single perpendicular incision, five or six inches long, commencing a little above the trochanter major, has been found to afford sufficient room for cutting out the head of the bone, where the parts are sound and free from morbid adhesion.

### Excision of the Knee-joint.

"The objections to excision of the knee-joint seem at first very great, and indeed insurmountable. It may be sufficient to mention the severity and danger of the operation, the tediousness of the cure, and the little difference as to utility between the stiff limb that is preserved and an artificial one. Upon closer examination, these objections, though they do not altogether vanish, certainly appear of less force. Thus the operation requires comparatively small superficial incisions, and can be accomplished much more quickly and easily than excision of the elbow-joint. It certainly must be regarded as more dangerous than amputation when the patient is very weak or exhausted by previous disease; but if he possesses moderate strength, I think it cannot be maintained, either on the general principles already stated, or from the result of experience, that the risk attending it is more than what proceeds from removing the limb. The recovery was certainly very tedious in Mr. Park's case, but there were particular circumstances which in some measure account for this; and the few patients who have since then undergone the operation recovered in a shorter time. It ought here to be recollected, too, that though recovery from amputation of the thigh is usually completed in three or four weeks, it is generally *at least as many months* before the patient can rest the weight of his body on the face of the stump, so as to use it in standing or walking. As to the utility of the limb, we find that it can be employed freely in progressive motion, and all the patients have declared that they consider themselves extremely fortunate in having preserved their legs such as they were. The advantages of the operation which may be contended for, are, that it preserves the natural support of the body afforded by the bones and joints of the *tarsus, metatarsus,* and toes, which, by diffusing the effects of force applied at the extremity of the limb, protects both it and the other parts of the body from concussion; and that it obviates the necessity of resting the whole of the patient's weight on the *face* of a stump, which must be done when amputation is performed above the knee. On the whole, I am not inclined to condemn the excision of the knee-joint altogether; and at the same time cannot venture to recommend it, without more facts to ascertain the correctness of our hypothetical opinions on the subject." 132.

Mr. Syme's plan of operating is to make two semilunar incisions across the fore-part of the joint, extending from one lateral ligament to the other, meeting at their extremities, and including the patella between them. If more room is required it may be obtained by cutting longitudinally at the point of union of the transverse incisions. The patient being laid on his back, the cavity of the joint should be rapidly opened and the patella removed. The lateral ligaments being next cut, the extremity of the femur may readily be protruded, and as much as seems necessary sawed off. The diseased part of the tibia can be easily taken away by passing the knife round the head of the bone, so as to detach its connexions, and then sawing off a slice of the requisite thickness. The popliteal vessels are little endangered; one or two of the articular branches may require to be tied. Great difficulty has been experienced in bringing the limb into a straight position after the operation, owing to the contracted state of the flexor muscles. The surgeon must be satisfied with placing the limb on a double-inclined plane, in as good a position as can be obtained by moderate force, exerted by pasteboard splints. In a few days the tension gradually diminishes, and before long the leg can be completely straightened. During the cure a slight degree of motion ought to be allowed, in order to prevent perfect osseous union. The chief difficulty consists in preventing the tendency to bend outwards, the best mode of opposing which consists in the careful application of splints. Two cases of excision of the knee-joint are dedailed. We shall notice them succinctly.

In the first case, that of a boy aged 8, the disease had existed three years, there were sinuses, the joint was much enlarged, and bent at an acute angle. The health was broken. The operation was performed on the 7th December. There was much difficulty encountered in consequence of the contraction of the ham-string tendons, and after the operation there was a great tendency to the tibia being drawn up behind the femur. At the end of some days this was counteracted, the limb became quite straight, and all did well. In four weeks after the operation, the wound was nearly healed, in three months it had regained so much strength that the patient could make some use of it in walking. At present he can walk and run, though with a halt, and merely requires the *heel* of the shoe to be two inches higher than the other. The limb is stout, slightly bowed outwards, and allows a slight degree of flexion and extension.

In the second case the operation was performed on the 14th December, and the boy died on the 8th of January.

### Excision of the Ankle-Joint.

The following observations on this operation are characterized by fairness and judgment.

"Next to the knee-joint the ankle is the most common seat of white-swelling, and the practicability of its excision is therefore an important subject of inquiry. It might be thought that the same objections would apply here as to the wrist-joint, but they hardly do so, at least to an equal extent. Instead of the three carpal bones which are connected with the radius and ulna, there is only one of the tarsus united with the tibia and fibula, viz. the astragalus, and it is of so large a size that the articular surface may be removed without encroaching on its connexions with the other tarsal bones; while in the wrist it is impossible to take away any portion of the carpal part of the articulation without opening other joints, and thus laying the foundation of future disease by exciting inflammation in a structure predisposed to unhealthy action. In the ankle, too, the tendons are less numerous, and the bones are of a larger size, so that more room can be obtained for their removal. But though excision of the ankle may thus be not so objectionable as that of the wrist, it cannot boast of much advantage. The object to be gained being merely a support for the body, it may be questioned how far the foot that remains after the ankle-joint has been cut out is superior for this purpose to an artificial one. It appears from the experience of Moreau, that anchylosis is very apt to ensue after the operation; and though, as he observes, the other joints acquire an unusual degree of mobility, so as to compensate in some measure for the rigidity which is thus caused, there can be no doubt that the elasticity of the foot will be greatly impaired. The limb, too, must be considerably shortened, and the ankle little calculated for bearing the severe strains to which it is exposed. It may be proper to notice also, that a very large proportion of the diseases usually referred to the ankle-joint are seated in the articulation between the astragalus and *os calcis*." 141.

Moreau's mode of operating appears to Mr. Syme to be the best. Two incisions, three inches in length, or more, are to be made along the posterior edges of the tibia and fibula, from their inferior extremities upwards; then from the lower ends of these two transverse cuts, in a direction forwards, as far as the tendon of the tibialis anticus on the tibial side, and to that of the peronæus tertius on the fibular side. The flaps being raised, the bones of the leg are exposed, and may be divided by the saw or pliers as high as necessary; after which the separation of their ligamentous connexions is easily effected. The articular surface of the astragalus may lastly be readily removed by the gouge or cutting pliers. The limb should be gently moved during the cure, to effect a firm fibrous union rather than an osseous one.

### Caries of the Tarsus and Metatarsus.

Attempts have been made to cut out the tarsal and heads of the metatarsal bones when carious. Unless the disease is confined to the os calcis, and removable without opening into the tarsal articulations, Mr. S. believes that the practice will be unsatisfactory. When the os calcis alone is affected the disease may be extirpated by making a crucial incision on the fibular side, and then digging out the carious part with a gouge.

"If the disease extends to any of the other tarsal or metatarsal bones, there is hardly any remedy but amputation; and if either the *astragalus* or *os calcis* be affected, of course the whole foot must be removed. It will be well, however, to recollect in such cases, that it is neither necessary nor useful to take away so much of the leg as is usually done. If the amputation is performed in a proper manner at or below the middle, so that a good cushion of muscle and integument is left to protect the extremities of the bones, the patient will retain the use of his knee-joint, and be able to stand or walk with an artificial foot, or short wooden pin, much more conveniently than he could do if obliged to support himself by resting on the knee. As the half of the leg is sufficient for this purpose, the surgeon should not amputate lower than this, since, though it may sometimes be possible to obtain a good stump by doing so, it much more frequently happens that, from the soft parts being too scanty in proportion to the size of the bones, these are badly covered." 144.

When both the os calcis and astragalus are sound, it is practicable to save an useful part of the foot by partial amputation.

### Partial Amputation of the Foot.

According to Mr. Syme's observations, the principal parts of the foot liable to be affected by caries are three;—the articulation between the bones of the leg and the astragalus—the articulation between the astragalus and os calcis—the further range of tarsal bones and neighbouring heads of the metatarsal. Here the disease is completely within reach of an amputation, such as Chopart's, which leaves only the os calcis and astragalus. Mr. Syme considers and meets the objections which have been urged against the operation, but we may omit his arguments, and content ourselves with shewing his method of operating.

"When the operation is to be performed, a tourniquet ought to be applied; and as it is always proper to compress vessels as near as possible to the part where they are to be divided, the pad should be placed over the posterior tibial artery, just above the ankle. Various minute directions have been given for determining the position of the joints. But the following very simple observation will, I am sure, be always found quite sufficient for the purpose. If the surgeon casts his eye upon the space between the outer ankle and head of the metatarsal bone of the little toe, he may easily ascertain the middle distance of these points; and this is the situation of the joint between the *os calcis* and cuboid bone. The other articulation, viz. that of the *astragalus* with the navicular, lies very nearly in the same transverse line; and the projection of the latter bone renders its discovery still more ready and certain. The flaps may be formed either entirely from the sole of the foot, or partly from it, and partly from the integuments of the instep; but the former plan is preferable, as affording a better covering for the bones. In this case it is necessary to make the flap extend fully to the balls of the toes, or farther extremities of the metatarsal bones; and it is here that one is most apt to go wrong, by cutting the parts too short for forming a good stump. In making the incisions, it is recommended to effect the disarticulation before making the flap from below; but I have uniformly found in operating on the dead subject, that it was extremely difficult in this way to cut it smoothly, owing to the relaxation of the parts in the sole of the foot, which ensues upon the separation of the bones. It is much better, therefore, to transfix the foot from side to side, and complete the section of the flap before opening the joints, while the parts are held steady under the knife. After this analysis of the operation, it may be well to give a connected account of the mode of performing it.

The surgeon, having recognized the position of the joints, should place the points of his thumb and fore-finger upon them, embracing the foot in his hand; then, with a small sharp-pointed amputating knife, blunt on the back, make an incision from the one to the other, slightly curved towards the toes, in order that it may correspond to the flap below; and next, instead of opening the joints, run his knife through from side to side, between the bones and flesh of the sole, and cut forwards close to the bones, until he arrives at the balls of the toes, when he terminates the incision, not abruptly, but gradually, so as to have a smooth edge and surface. Nothing now remains but the disarticulation, which may be effected with extreme facility, as the surfaces of the joints are nearly straight, and in the same line.

The anterior tibial and external plantar arteries require to be tied; after which the flap being stitched into its place, compresses and a bandage ought to be applied. The limb during the cure ought to be kept bent to relax the gastrocnemius." 153.

Two cases are given in illustration, but these need not be noticed. We have now done with the present work, and a few words on parting are all that remain. We think that the facts which are detailed by Mr. Syme are sufficient to prove that excision of joints is much more generally useful and advisable than was imagined. We cannot say, but we think that Mr. S. is inclined to go a little too far, but this is a matter of opinion, and we may be wrong. We believe that the operation will come into more or less general use, and then the profession will have the best, indeed the only satisfactory evidence of its merits or defects. It appears to be more applicable to the upper extremity than to the lower, and of all the articulations it seems most adapted to that of the shoulder. Within these few weeks the head of the humerus has been twice excised at St. George's Hospital for caries and disease of the shoulder-joint. Both the patients are doing well.

A word or two on the author, and on the execution of his book. Mr. Syme seems generally actuated by a spirit of candour and fairness in his observations, and he evinces very little prejudice or bias in favour of the views which he more immediately supports. The work is not a model of composition, but that is not to be expected. It is likely to prove useful in the hands of those who may be destined to perform operations, and we suppose that utility is Mr. Syme's chief aim.

